# Ectopic parathyroid gland within carotid sheath

**DOI:** 10.1002/ccr3.3476

**Published:** 2020-11-22

**Authors:** Benjamin Ruimin Poh, Rahul Harshad Nagadia, Gerald Ci‐An Tay

**Affiliations:** ^1^ Singhealth Duke‐NUS Head and Neck Centre Singapore General Hospital Singapore Singapore

**Keywords:** endocrine surgery, general surgery, parathyroid

## Abstract

During operative exploration of the neck for parathyroid surgery, the surgeon should always consider possible ectopic locations of the glands and have a reasonable surgical strategy for locating these ectopic glands.

## Graphical abstract

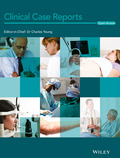

This 35‐year‐old patient had previous left adrenalectomy for nonfunctioning adrenal adenoma, excision of his right inferior parathyroid gland for parathyroid adenoma 6 years ago and open enucleation of pancreatic head and uncinate process neuroendocrine tumors (insulinomas) 2 years ago. He now presents with recurrent hypercalcaemia secondary to primary hyperparathyroidism and operative exploration of his left neck reveals the following image.

What clinical syndrome does he fulfill, and what are the lesions presented in the given image?

This patient tested positive for a mutation in his multiple endocrine neoplasia type 1 (MEN1) gene.^1^ On operative exploration of his neck, he was found to have had four remnant hyperplastic parathyroid glands (including one supernumerary gland present in his cervical thymus). The operative image demonstrates both his left parathyroid glands (Figure 1), with the superior gland (yellow arrow) in the prevalent position^2^ posterior to the left superior thyroid pole (thyroid gland retracted medially by Allis forceps), and the inferior gland (blue arrow, also grossly hyperplastic) in an ectopic position located within the carotid sheath, adjacent to his visualized vagus nerve (orange arrow) and carotid artery (laterally).

**FIGURE 1:**
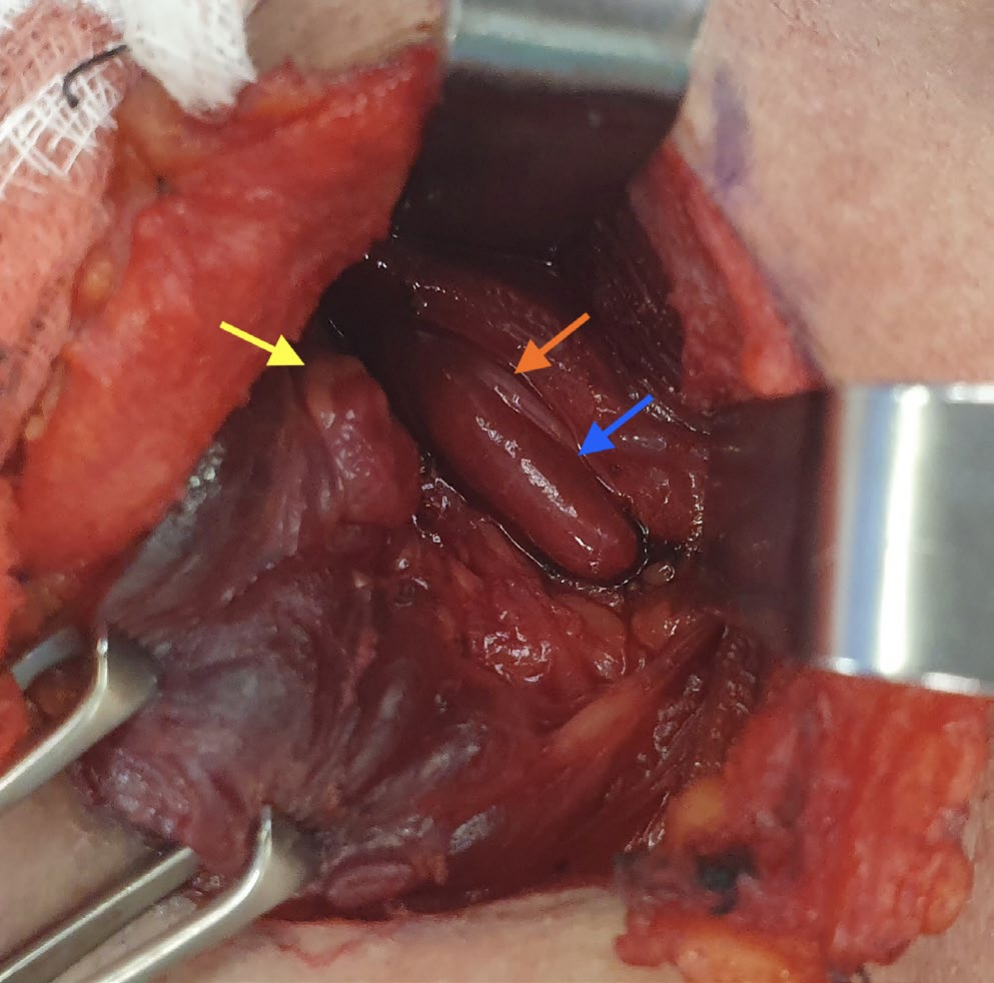
Ectopic inferior parathyroid gland located within the carotid sheath (blue arrow), see text for more details

In the case of possible missing or ectopic parathyroid glands, we recommend a logical approach to dissection of these glands intra‐operatively starting from the most likely and accessible areas to least so. For superior glands, the posterior surface of the upper pole of the ipsilateral thyroid gland should be carefully inspected, following which a search can be undertaken along the tracheo‐oesophageal groove, para‐oesophageal space, posterior retro‐oesophageal space, and piriform sinus. In the case of inferior glands, the search should follow the thyro‐thymic tract to the thymus (anterior mediastinum) wherein a cervical thymectomy may be performed (especially for patients with known MEN syndrome), subsequently the carotid sheath may be dissected (carefully preserving the vagus nerve), failing which an ipsilateral thyroid lobectomy may be performed if suspicion for an intrathyroidal gland exists.^1^, ^2^

## CONFLICT OF INTEREST

None declared.

## AUTHOR CONTRIBUTION

BP, RN, GT: contributed equally to the preparation, editing, and revision of this manuscript.

## ETHICAL APPROVAL

Patient consent was sought for the production of this article, and no Ethics Committee approval was required for this publication.

## Data Availability

The data that support the findings of this study are available on request from the corresponding author. The data are not publicly available due to privacy or ethical restrictions.
